# Prevalence of contraindicated combinations amid behavioral and mental health medications filled in a pediatric population

**DOI:** 10.1186/s12875-024-02528-9

**Published:** 2024-07-30

**Authors:** Laura M. Borgelt, Kathryn Bliss, Jacqueline Matson, Bosede Cajuste, Xiaoying Kuang, Monica Toohey, Wilson Pace, Eyal Shemesh, Suzanne Lo, Anna Olczyk, Kristine Gleason, Harold Pincus, Lawrence C. Kleinman

**Affiliations:** 1 University of Colorado Anschutz Medical Campus, 1890 N Revere Ct., Mailstop L606, Aurora, CO 80045 USA; 2 New York State Department of Health, Albany, NY USA; 3 DARTNet Institute, Aurora, CO USA; 4 Department of Pediatrics, Icahn School of Medicine at Mount Sinai, New York City, NY USA; 5 Case Western Reserve University, Rainbow Babies & Children’s Hospital, Cleveland, OH USA; 6 Advent Health, Hinsdale, IL USA; 7 New York Presbyterian Hospital, Columbia University, New York City, NY USA; 8 Rutgers Robert Wood Johnson Medical School, New Brunswick, NJ USA

**Keywords:** Adolescents, Behavioral medicine, Drug combinations, Drug interactions, Medicaid, Mental health, Pediatrics, Polypharmacy

## Abstract

**Background:**

Behavioral or mental health disorders are common in children, adolescents, and young adults. Medication use is increasingly common, with few data describing drug-drug combinations in ambulatory settings. The objectives of this study were to describe the pharmaco-epidemiology of behavioral and mental health (BMH) medications among children, adolescents, and young adults in New York Medicaid and assess the prevalence of contraindicated drug pairs within this population.

**Methods:**

This observational cross-sectional study evaluated New York State Medicaid managed care and fee-for-service enrollees under 21 years of age dispensed BMH medications in 2014. Main outcomes included number of members with prescriptions filled; number filling > 1 medication prescription concurrently for ≥ 30 days (polypharmacy), and number and nature of potentially contraindicated drug pairs.

**Results:**

Of 2,430,434 children, adolescents, and young adults, 422,486 (17.4%) had a visit associated with a BMH diagnosis and 141,363 (5.8%) received one or more BMH medications. With 84 distinct medications evaluated, polypharmacy was common, experienced by 53,388 individuals (37.8% of those with a prescription filled), generating 11,115 distinct drug combinations. 392 individuals filled prescriptions for a contraindicated pair of ≥ 2 BMH medications for 30 days or longer. With ≥ 1 day overlap, 651 were exposed to contraindicated medications. The most common contraindicated pairs increased potential risk for prolonged QT interval and serotonin syndrome (*n* = 378 and *n* = 250 patients, respectively). Most combinations involved ziprasidone (3247.1 per 10,000 ziprasidone prescriptions filled).

**Conclusions:**

With nearly 6% of members dispensed a BMH medication, contraindicated drug pairs were uncommon. However, any of those combinations represent a potential risk. Clinicians should attend to the balance of potential risks and benefits before contraindicated pairs are dispensed. The methodology described could serve as a basis for monitoring such rare instances and might reduce harm.

**Supplementary Information:**

The online version contains supplementary material available at 10.1186/s12875-024-02528-9.

## Background

The increasing prevalence of psychiatric diagnoses and hospital admissions in children and young adults correlates with the increasing number of prescriptions and complexity for behavioral or mental health (BMH) medications and polypharmacy in the pediatric population [1–6]. From the 1990s to the early 2000s, prescription rates for psychostimulants, antipsychotics, and antidepressants doubled [4]. These rates have continued to climb and have even outpaced increases seen in adult prescription rates over the past two decades [5, 7, 8]. Concurrent use of multiple medications or polypharmacy has been cited as a serious risk factor for the development of adverse drug reactions in the pediatric population presumably as a result of exposure to drug-drug interactions (DDIs) [9–11]. One might think that children would be at a decreased risk for polypharmacy; however, children taking psychotropic medications are at a higher risk of using multiple medications compared with those that are of advanced age [7]. Additionally, most adults and children on BMH medications receive their prescriptions from a primary care physician and not a psychiatrist who specializes in prescribing BMH medications [8].

Girand et al. evaluated 121,481 ambulatory care visits for patients aged 2–24 years and found attention-deficit/hyperactivity disorder (ADHD) medication prescribing increased from 4.8 to 8.4%, ADHD polypharmacy increased from 16.8 to 20.5%, and psychotropic polypharmacy increased from 26.0–40.7%.^7^ These findings may have overestimated actual medication use because the data only reflected rates of prescribing at ambulatory visits and did not account for adherence to therapy or short-term therapeutic overlap. Burcu et al. noted that antipsychotics were commonly co-prescribed with one other psychotropic medication class in half (50.7%) of 4,603 behavioral disorder visits, and with two or more other psychotropic medication classes in 39.1% of these visits [8]. These results showing increased rates of polypharmacy with BMH medications, specifically concurrent use of two medications in this population, warrants further evaluation of any concerning drug pairs [12].

This study set out to advance the understanding of commonly prescribed BMH medications in children, adolescents, and young adults in the New York State (NYS) Medicaid population by (1) assessing characteristics of those dispensed BMH medications and (2) determining the prevalence of contraindicated drug pairs (CDPs) in the outpatient setting.

## Methods

### Design and population

This observational cross-sectional study includes NYS Medicaid managed care (MMC) and fee-for-service (FFS) enrollees under 21 years of age who received any Medicaid services in 2014 that included a BMH diagnosis or filled a prescription for a medication that was considered a BMH medication. This study was approved by the Institutional Review Board and our detailed methods are described in Appendix A. We used ICD 9 codes and well accepted schema to identify children with BMS diagnoses and we used National Drug Code (NDC) codes and HEDIS^®^ medication lists to identify BMH medications [13, 14]. Our primary data source was NYS 2014 Medicaid pharmacy plus fee-for-service (FFS) claim and Medicaid Managed Care (MMC) encounter data, excluding those children eligible for both Medicaid and Medicare and those that did not receive any services. In 2014, 2,430,434 million persons under 21 years were enrolled in NYS Medicaid and 2.1 million received services. Demographic information was gleaned from Medicaid enrollment data. Chi square contingency tables were used to determine the distribution of patient characteristics in enrollees with BMH medications compared to enrollees with CDPs and to establish *P*-values.

The Medicaid drug database included a reference table with all NDCs rolled up to a generic drug name. In 2014, 1.6 million enrollees (65.8%) filled at least one prescription. When searching for CDPs, overlaps in NDCs that rolled up to generic drug names on the contraindicated list were evaluated. Concurrent use of contraindicated drugs was defined as overlapping fills of two or more contraindicated BMH medications for at least thirty days, allowing for a possible 32-day gap between consecutive start and end dates of the same medication. Fixed-combinations were treated as one medication. Injectable meds were included (e.g., haloperidol decanoate). IBM Micromedex was used to identify which drug pairs were contraindicated [15].

All BMH medications were considered for the primary analysis of assessing characteristics of medication dispensed and the potential for dispensing CDPs. Secondary analyses included more in-depth evaluation of medication(s) with significant clinical implications and clinicians who prescribed CDPs that were filled. Given the high number of prescriptions filled and CDPs that included ziprasidone, a post-hoc analysis was conducted. We identified prescribers of BMH medications, including CDPs, from pharmacy claims with clinician specialty extracted from the Medicaid database of NYS Medicaid providers. We excluded CDPs when both medications were not BMH medications. This study focuses on contraindicated interactions and excludes even moderate and major interactions, which may require changes in therapy or be life-threatening, respectively.

## Results

### Patient population

In 2014, 422,486 enrollees had an encounter associated with a BMH diagnosis; and 5.8% of all enrolled (141,363) filled a prescription for BMH medication whether-or-not they had evidence of a BMH diagnosis. (Table 1) As shown in Appendix B, the most commonly prescribed BMH medications were methylphenidate, dextroamphetamine/amphetamine, and clonidine; however, none of these medications were paired with contraindicated medications. CDPs occurred in 0.3% (*n* = 392) of patients dispensed BMH drugs.

**Table 1:** Characteristics of 141,363 members (under 21 years) enrolled in New York State Medicaid (MMC & FFS) in 2014 dispensed at least one behavioral or mental health medication compared with those that were dispensed a contraindicated drug pair

Members enrolled in New York State Medicaid in 2014 with at least one behavioral or mental health medication filled but without contraindicated drug pairs (*n* = 140,971)			Members enrolled in New York State Medicaid with dispensed contraindicated drug pairs (30-day concurrency) (*n* = 392)		*P*-Value
**Characteristic**	Frequency	% of Enrollees	Frequency	% of Enrollees	
**Gender**					0.0051
Male	87,026	61.7	215	54.9	
Female	53,945	38.3	177	45.2	
**Age Category**					< 0.0001
0–5 yr	5,198	3.7	0	0	
6–11 yr	50,497	35.8	37	9.4	
12–17 yr	58,905	41.8	203	51.8	
18–20 yr	26,371	18.7	152	38.8	
**Foster Status**					0.6309
Other	135,261	96.0	378	96.4	
Foster Child	5,710	4.1	14	3.6	
**Institutionalized**	843	0.6	15	3.8	< 0.0001
Urbanicity*					0.2862
Large City	90,488	64.2	229	58.4	
Small City	29,958	21.3	91	23.2	
Non-Urban	19,682	14.0	57	14.5	
Region*					< 0.0001
Central	20,190	14.3	65	16.6	
Hudson Valley	12,805	9.1	42	10.7	
Long Island	10,306	7.3	40	10.2	
Northeast	18,201	12.9	42	10.7	
NYC	47,347	33.6	87	22.2	
Western	31,279	22.2	101	25.8	
**Medicaid Eligibility**					< 0.0001
Non-SSI**	101,681	72.1	174	44.4	
SSI	39,290	27.9	218	55.6	
**Insurance Type**					< 0.0001
Fee-for-service	36,790	26.1	151	38.5	
Medicaid managed care	104,181	73.9	241	61.5	
**Race/Ethnicity**					0.0002
White	64,918	46.1	223	56.9	
Black	22,561	16.0	58	14.8	
Asian	3,214	2.3	Masked	1.0	
Hispanic	21,805	15.5	52	13.3	
Other/Unknown	28,473	20.2	55	14.0	
**Cash Assistance**					< 0.0001
None	89,793	63.7	196	50.0	
Cash Assistance	51,178	36.3	196	50.0	
**Behavioral or Mental Health Diagnosis**					< 0.0001
Yes	18,332	13.0	374	95.4	
No	122,639	87.0	18	4.6	

To protect anonymity, counts less than 6 have been suppressed. To prevent calculating suppressed values, the second smallest values in a category have been masked. If masked, rates were calculated assuming the value of the suppressed cell = 5

*Non-institutionalized

**Non-SSI includes Aliens, Safety Net, and Unassigned

MMC: Medicaid managed care

FFS: Fee-for-service

During the study year (2014), all claims, including the encounter/claim with the BMH medication, were reviewed for a BMH diagnosis. Among those without a CDP, 87% did not have an associated BMH diagnosis, most likely because they received a diagnosis in a previous year. This information was not analyzed because not all children were enrolled in NYS Medicaid in previous years. The ten most common BMH diagnoses accounted for more than 60% of all BMH diagnoses. These included attention deficit disorder with hyperactivity; mixed receptive-expressive language disorder; unspecified delay in development; anxiety state, unspecified; other developmental speech or language disorder; depressive disorder, not elsewhere classified; counseling on substance use and abuse; autistic disorder, current or active state; unspecified disturbance of conduct; and oppositional defiant disorder. In the 18,332 members with both a BMH diagnosis and BMH medication, the most common diagnoses were very similar to the larger group of members with BMH diagnoses. The only differences found between these groups were that among the group with a BMH medication and BMH diagnosis, language and developmental delays were less common and depression, anxiety, and posttraumatic stress were slightly more common.

Table 1 illustrates differences between the cohort of NYS Medicaid members dispensed a BMH medication with and without a CDP. Gender distribution was almost 2:1 (male: female) in those without a CDP, but nearly equal for those dispensed a CDP, (*p* = 0.0051). Those who filled prescriptions for a CDP were older (*p* < 0.0001), more likely to reside outside NYC (*p* < 0.0001), more likely to be white (*p* = 0.0002), more likely to have Medicaid SSI eligibility status (*p* < 0.0001), more likely to require cash assistance (*p* < 0.0001), and more likely to have a BMH diagnosis (*p* < 0.0001) than those without a CDP. Among those with CDP, a BMH diagnosis was reported in 95% of the cases (vs. 13% in subjects without CDP).

Of note, there are observed demographic differences in the children who are prescribed ziprasidone without a CDP compared to those prescribed ziprasidone with a CDP. Most children in the CDP group were prescribed ziprasidone, so certain characteristics of this group may be skewed by the patient characteristics associated with ziprasidone prescribing, such as age; however, these differences could also be related to other factors.

### Filled prescriptions

Eighty-four distinct BMH medications were filled for Medicaid enrollees under age 21 in 2014 and evaluated. Polypharmacy was common among patients dispensed BMH medications as 37.8% (*n* = 53,388) received two or more BMH medications with 30 days or more concurrency (≤ 32-day gap allowed) generating 11,115 distinct drug combinations. Removing the requirement for 30 days of concurrency to 1 day or more concurrency increased the number experiencing polypharmacy to 44.5% (*n* = 62,945) with 19,711 distinct drug combinations. Patient consumption of the medications could not be assessed.

### Contraindicated drug pairs

**Fig. 1:**
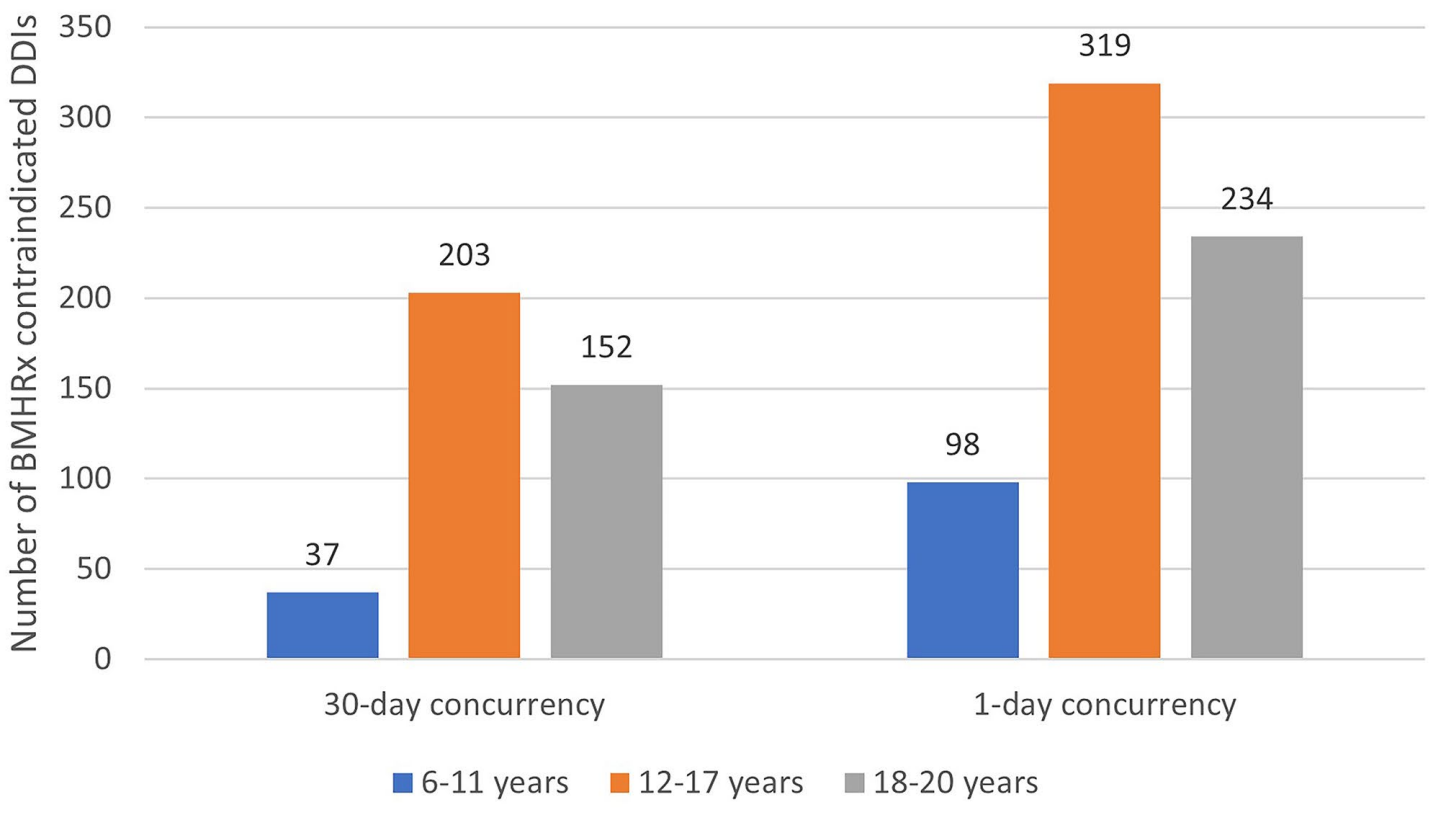
Number of persons with potential contraindicated drug pairs dispensed by age group and concurrency

A total of 392 children, adolescents, and young adults were dispensed contraindicated pairs of BMH medications for at least 30 overlapping days. (Table 2) This table shows the number of patients prescribed any specific medication and since they were often prescribed more than 1 BMH medication, children were counted multiple times. The total number of distinct children impacted by this prescribing (141,363) and the CDPs (392) are shown at the bottom of the table. Of 84 BMH filled prescriptions assessed, 24 medications were a part of at least one CDP. Figure 1 shows the number of CDPs used in this population stratified by age and consider both 30 days of concurrency and any overlap of at least 1 day. A higher number of patients ages 12–17 years were dispensed CDPs than younger (6–11 years) and older (18–20 years) patients at both concurrency time points. No children ages 0–5 years were dispensed a contraindicated DDI.

**Table 2:** Frequency of BMH prescriptions filled and prevalence for contraindicated drug pairs among BMH medications with at least 30 days concurrency.*

BMH Drug	# of members (< 21 years) with BMH prescription filled in 2014	# of members (< 21 years) with a contra-indicated drug pair	# of members with contra-indicated drug pair age 6–11 years	# of members with contra-indicated drug pair age 12–17 years	# of members with contra-indicated drug pair age 18–20 years	Contraindicated pair rate per 10,000 persons members with any BMH medications prescription filled	Contraindicated pair rate per 10,000 members with the BMH prescription filled
**Ziprasidone hydrochloride**	1121	364	37	191	136	25.7	3247.1
**Fluoxetine hydrochloride**	10,626	96	11	48	37	6.8	90.3
**Trazodone hydrochloride**	4594	76	9	43	24	5.4	165.4
**Quetiapine fumarate**	8778	43	Masked	23	Masked	3.0	49.0
**Risperidone**	18,741	41	8	14	19	2.9	21.9
**Aripiprazole**	8616	36	Masked	23	Masked	2.5	41.8
**Citalopram hydrobromide**	5451	35	Masked	17	Masked	2.5	64.2
**Escitalopram oxalate**	4697	33	0	19	14	2.3	70.3
**Olanzapine**	2667	32	Masked	16	Masked	2.3	120.0
**Venlafaxine hydrochloride**	1412	16	0	Masked	10	1.1	113.3
**Chlorpromazine hydrochloride**	803	16	Masked	10	Masked	1.1	199.3
**Carbamazepine**	1418	13	0	Masked	Masked	0.9	91.7
**Lurasidone hydrochloride**	405	13	0	Masked	Masked	0.9	321.0
**Paroxetine hydrochloride**	1514	11	Masked	Masked	< Masked	0.8	72.7
**Other**	15,369	41	Masked	24	Masked	2.9	26.7
**Total distinct children**	**141**,**363**	**392**	**37**	**204**	**152**	**27.7**	**27.7**

*Persons may take > 1 BMH medication and therefore a patient may be counted in multiple categories

To protect anonymity, counts less than 6 have been suppressed. To prevent calculating suppressed values, the second smallest values in a row have been masked

If < 6, rates were calculated using *n* = 5

BMH = Behavioral or Mental Health

### Contraindicated drug pairs based on concurrency

As expected, the rate of prescribed CDPs decreased when we increased the necessary days of concurrency. Table 2 presents data for a 30-day minimum threshold for concurrency for members prescribed BMH medications. Using the 30 days threshold for concurrency, the overall rate of contraindicated drug combinations was 27.7 per 10,000 members (actual rate 0.00277) who filled at least one BMH prescription. Nearly one-third of CDPs occurred with ziprasidone (32.4%, 364/1121 members). Rates of CDPs per 10,000 members were highest for ziprasidone (25.7), fluoxetine (6.8), and trazodone (5.4).

Changing to a minimum of 15 days of concurrent supply of medication, 514 members were dispensed a contraindicated drug combination, a rate of 36.4 per 10,000 persons filling BMH prescriptions (actual rate 0.00364). Using this threshold, thioridazine was associated with a CDP half of the time (9/18 children, 50.0%) followed by ziprasidone (42.3%, 474/1,121 children). Rates of contraindicated DDIs per 10,000 members were highest for ziprasidone (33.5), fluoxetine (7.6), and trazodone (6.5).

Further reducing the threshold to 1 day or more increased the members with a BMH medication filled experiencing CDP to 651 members (46.1 per 10,000 or an actual rate of 0.00461). Using this most stringent definition, 11 of 18 children (61.1% or 6,111 per 10,000) dispensed thioridazine and 606 of 1,121 children (54.1% or 5,406 per 10,000) dispensed ziprasidone were associated with contraindicated pairs of medications. Rates of CDPs per 10,000 children were highest for ziprasidone (42.9), fluoxetine (8.6), risperidone (8.3) trazodone (7.4), and quetiapine (7.2).

### Specific contraindicated drug pairs and clinical concerns

Table 3 provides data specific to the CDPs. The most common CDPs involved ziprasidone prescribed with fluoxetine (*n* = 94), trazodone (*n* = 76), quetiapine (*n* = 40), risperidone (*n* = 39), aripiprazole (*n* = 34), citalopram (*n* = 33), and escitalopram-(*n* = 33). Days of concurrency in the study year ranged from 30 to ≥ 360 days. Though not shown in Table 3 due to low counts (< 10 persons), pimozide had CDPs with concurrent aripiprazole, citalopram, chlorpromazine, fluoxetine, olanzapine, risperidone, and sertraline. Thioridazine was prescribed concurrently with citalopram, olanzapine, quetiapine, risperidone, and venlafaxine.

**Table 3:** Characteristics of contraindicated drug pairs among members 21 years and younger with at least 30 days of concurrency (*n* = 392)

Contraindicated drug pairs and documentation status*	Number of members with concurrent use of contraindicated drug pair	Rate per 10,000 members with a BMH prescription filled	Minimum number of days of concurrent use	Maximum number of days of concurrent use	Average number of days of concurrent use
**Ziprasidone hydrochloride**	364	3247.1	30	360	
Ziprasidone hydrochloride—fluoxetine hydrochloride (Fair)	94	838.5	30	360	110
Ziprasidone hydrochloride—trazodone hydrochloride (Fair)	76	678.0	30	360	92
Ziprasidone hydrochloride—quetiapine fumarate (Fair)	40	356.8	30	352	113
Ziprasidone hydrochloride—risperidone (Fair)	39	347.9	30	344	101
Ziprasidone hydrochloride—aripiprazole (Fair)	34	303.3	30	343	115
Ziprasidone hydrochloride—citalopram hydrobromide (Fair)	33	294.4	30	353	117
Ziprasidone hydrochloride—escitalopram oxalate (Fair)	33	294.4	30	344	118
Ziprasidone hydrochlorisde—olanzapine (Fair)	30	267.6	30	339	81
Ziprasidone hydrochloride—chlorpromazine hydrochloride (Fair)	15	133.8	30	354	129
Ziprasidone hydrochloride—paroxetine hydrochloride (Fair)	11	98.1	30	337	93
Ziprasidone hydrochloride—clomipramine hydrochloride (Fair)	Masked	Masked	34	330	137
Ziprasidone hydrochloride—clozapine (Fair)	Masked	Masked	31	181	106
Ziprasidone hydrochloride—doxepin hydrochloride (Fair)	Masked	Masked	37	118	87
Ziprasidone hydrochloride—haloperidol (Fair)	Masked	Masked	30	338	131
Ziprasidone hydrochloride—imipramine hydrochloride (Fair)	Masked	Masked	30	267	146
Ziprasidone hydrochloride—paliperidone (Fair)	Masked	Masked	74	265	170
Ziprasidone hydrochloride—perphenazine (Fair)	Masked	Masked	30	229	73
Ziprasidone hydrochloride—venlafaxine hydrochloride (Fair)	15	133.8	30	343	115
**Carbamazepine**	**13**	**91.7**	**30**	**309**	
Carbamazepine—lurasidone hydrochloride (Good)	13	91.7	30	309	125
**Lurasidone hydrochloride**	**13**	**321.0**	**30**	**309**	
Lurasidone hydrochloride—carbamazepine (Good)	13	321.0	30	309	125
**Others (all < 10 persons)**	**Masked**	**Masked**	**Masked**	**Masked**	**Masked**

*Documentation definition of “fair” is “available documentation is poor, but pharmacologic considerations lead clinicians to suspect the interaction exists; or, documentation is good for a pharmacologically similar drug.” Documentation definition of “good” is “documentation strongly suggests the interaction exists, but well-controlled studies are lacking.”

Total counts include all potential contraindicated drug pairs, but not all data for combinations are shown in table to protect anonymity for counts less than 10 persons

Given the high number of prescriptions filled and CDPs that included ziprasidone, a post-hoc analysis revealed numerous BMH diagnostic classes associated with persons that have CDPs with ziprasidone. Almost all, (95.6%, 348/364) persons that had CDP including ziprasidone had a BMH diagnosis, with 90% having two or more BMH diagnoses. Ziprasidone was most frequently paired with a contraindicated drug for mood disorders (*n* = 229), ADHD (161), and anxiety (143). Specific BMH diagnoses include attention deficit disorder with hyperactivity (143), unspecified episodic mood disorder (132), and bipolar disorder, unspecified (123). Of 16 children that were prescribed CDPs including ziprasidone without a BMH diagnosis, the most common diagnosis codes recorded were for unspecified morbidities, well-child visits, and vaccinations, suggesting that our strategy missed relevant diagnostic information.

The identified CDPs presented serious risk (Table 4). The most common potential drug interactions among dispensed BMH medications risk potentially lethal cardiac dysrhythmias due to prolongation of the QT interval (*n* = 378 patients with at least 30-day concurrency). The second most common potential drug interactions among CDPs are associated with a risk of serotonin syndrome (*n* = 250 patients with at least 30-day concurrency), also potentially lethal.

**Table 4:** Frequency of potential contraindicated drug pairs with QT prolongation and serotonin syndrome as a possible (not actual) outcome for ≥ 10 persons

Contraindicated Drug-Drug Combination	# of members with contraindicated drug pair (≥ 1-day concurrency)	# of members with contraindicated drug pair (≥ 15-day concurrency)	# of members with contraindicated drug pair (≥ 30-day concurrency)
**QT prolongation**	628	492	378
Ziprasidone hydrochloride—fluoxetine hydrochloride	116	104	94
Ziprasidone hydrochloride—trazodone hydrochloride	103	91	76
Ziprasidone hydrochloride—risperidone	111	64	39
Ziprasidone hydrochloride—quetiapine fumarate	99	63	40
Ziprasidone hydrochloride—aripiprazole	91	57	34
Ziprasidone hydrochloride—olanzapine	64	43	30
Ziprasidone hydrochloride—escitalopram oxalate	50	44	33
Ziprasidone hydrochloride—citalopram hydrobromide	50	39	33
Ziprasidone hydrochloride—venlafaxine hydrochloride	31	26	15
Ziprasidone hydrochloride—chlorpromazine hydrochloride	27	18	15
Ziprasidone hydrochloride—haloperidol	22	16	Masked
Ziprasidone hydrochloride—paroxetine hydrochloride	19	16	11
**Serotonin Syndrome**	**341**	**306**	**250**
Ziprasidone hydrochloride—fluoxetine hydrochloride	116	104	94
Ziprasidone hydrochloride—trazodone hydrochloride	103	91	76
Ziprasidone hydrochloride—escitalopram oxalate	50	44	33
Ziprasidone hydrochloride—citalopram hydrobromide	50	39	33
Ziprasidone hydrochloride—venlafaxine hydrochloride	31	26	15
Ziprasidone hydrochloride—paroxetine hydrochloride	19	16	11

Total counts include all potential contraindicated combinations, but not all combinations are shown in table to protect anonymity for counts less than 10 persons

### Clinician prescribing

We identified 20,656 clinicians who prescribed the 84 BMH medications included in our analysis. There were 386 clinicians that prescribed at least one contraindicated pair of medications (with 30-day concurrency), with a mean of 1.6 contraindicated pairs per these prescribers. More than one-third (37.8%) of these prescribers prescribed more than one contraindicated pair, with 23.8% prescribing two pairs, 8.3% prescribing three pairs, and 2.9% prescribing four distinct contraindicated combinations of two or more drugs. Another 2.9% prescribed more than four CDP. Given the significance of ziprasidone in this analysis, we identified 477 clinicians who prescribed this medication. For clinicians whose specialty was known by Medicaid (80.1%, 309/386), 65% were psychiatrists, and 5% were pediatricians. This difference may be related to psychiatrists seeing more severely ill, more treatment resistant patients than others.

## Discussion

This analysis from 2014 provides useful information as a pre-pandemic benchmark about prescription patterns (which may or may not have changed), especially as the use of psychotropic medications and psychotropic polypharmacy has increased in recent years [10, 11]. There are several ways to view our findings: it may be considered good news that there were a limited number of CDPs (< 1%) dispensed in this large Medicaid population. It is reassuring that the most commonly prescribed medications including methylphenidate, dextroamphetamine/amphetamine, and clonidine were not paired with medications that were associated with CDPs. Still, CDPs put children, adolescents, and young adults at significant risk for harm and sometimes represent what patient safety advocates might call “never events.” [16]. In this study, 392 patients filled a contraindicated combination of BMH medications with an overlap of 30 or more concurrent days. This number increased when the concurrency threshold was relaxed ( > = 15 days = 514, and > = 1 day = 651 patients). Because interactions can occur during transition periods, the monthly timeframe ensures that the potential for harm is not missed even when the time period is limited. We recognize that shorter durations (< 30 days) of overlap may occur as medications are titrated during transition periods and could lead to misclassifications of CDPs so they are not the primary time frame of reference. Even though this may be considered acceptable practice, CDPs during such transitions may still cause bad outcomes. Inclusion of this sensitivity analysis is supported by the suggested clinical practice definition of pediatric polypharmacy as “the prescription or consumption of two or more distinct medications for at least one day.” [12].

In this study, the frequency and rate of CDPs were greater in the adolescent and young adult than younger populations. A majority of CDPs included ziprasidone with prolongation of the QT interval as the main concern. Ziprasidone as a monotherapy has a potential risk of QT interval prolongation [17]. Because of this risk, the FDA has warned that ziprasidone should not be prescribed with other drugs that have demonstrated QT prolongations, as an additive impact cannot be ruled out. Our findings therefore support adherence to the recommendations for ziprasidone treatment which may include baseline potassium and magnesium measurements and/or electrocardiogram measures [18, 19]. This drug has the further disadvantage of increasing the risk of serotonin syndrome, a second potentially devastating complication. Given that many of the CDPs occurred with ziprasidone, it may be prudent to recommend greater attention to drug interactions with prescriptions of this agent, perhaps via medication monitoring or decision support.

Such rare but devastating consequences support both the study of potential drug interactions and consideration of the use of contraindicated pairs as indicators of high-risk prescribing with potential negative effects. We recognize that some use of these risky pairs may be undertaken after risks are carefully considered and determined to be beneficial in complex individual situations. There may not be less risky or evidence-based therapy available. Our perspective is that in such cases, good practice demands that patients and caregivers need to be informed of and assent to risks and benefits, including informing them of the fact that the intended pair of medications is contraindicated, or recommended against using in combination. The outpatient setting, as described in this study, should allow for careful evaluation of all BMH medications dispensed by all clinicians to ensure, at a minimum, that CDPs are avoided. Major drug interactions are not desirable but are an acceptable risk in specific circumstances.

One important limitation of this study is that our findings span one year of time. While prescribing patterns were likely reflective of this observational study until the pandemic, recent data demonstrate that the average days’ supply of psychotropic medications dispensed increased significantly throughout the pandemic [20]. Given this increase, our results point to the relevance of these data and the importance of ongoing monitoring of CDPs. Additionally, although medications are prescribed, it does not mean that they are taken by the patient which may reduce the true risk. Further, we do not include all sources of risk, such as over-the-counter and non-BMH medications. Some clinicians in this population may make clinical decisions that are supported in the literature (but not drug interaction databases) or take into consideration the risk-benefit of drug interactions. It is difficult to determine from this analysis the intent of the clinician and how and when clinicians are making considered judgments based on full information and appropriate engagement of patients. We have based our findings upon Micromedex, a respected and widely employed compendium, but other resources exist and may describe interactions differently. We have restricted our analysis to contraindicated drug pairs as articulated by Micromedex. There may be many other important drug interactions that we do not identify in this study. The context of these findings ought to be in a lower than typical risk environment since NYS Medicaid already includes measures to ensure appropriate prescribing of antipsychotics and other medications through drug utilization review programs. These reviews are focused on appropriate age, dosing, and frequency for single agents or classes, and alert pharmacists to potential DDIs. These interactions may be over-ridden at the pharmacy, after consultation. Our data are based upon risk and not actual harm, a common limitation in studies of rare events. Outcomes studies could be an important area of future research. In NYS, nearly half of all children are in Medicaid. This study, while based on NYS Medicaid and therefore mostly generalizable to underserved populations and perhaps beyond, may be an underestimate for populations not subjected to the utilization review and scrutiny of antipsychotic prescribing employed by NYS Medicaid.

## Conclusion

We found that nearly 6% of children, adolescents, and young adults filled a BMH medication and 392 were dispensed a contraindicated combination. Although infrequent, the use of contraindicated medication combinations could increase risk for adverse events. The fact that only a small (and therefore manageable) minority of children receive such prescriptions provides reassurance that monitoring for such prescription patterns, done at the level of the health maintenance organization, would not be onerous or lead to extensive warnings, but rather is likely to identify a very small number of real concerns, and help inform a granular review with the prescriber. We believe that our data and this methodology could serve as the beginning of such efforts. Such ongoing monitoring could lead to the identification of common medications that are more likely to be implicated, and therefore to increased efficiency in flagging such cases. Ultimately, such ongoing monitoring could lead to reductions in harmful prescription combinations and reduce the rate of medication-related adverse events.

### Electronic supplementary material

Below is the link to the electronic supplementary material.

Supplementary Material 1

## Data Availability

Data is provided within the manuscript or supplementary information files.

## Abbreviations

ADHD: Attention-deficit/hyperactivity disorder
BMH: Behavioral and mental health
CDP: Contraindicated drug pairs
DDI: Drug-drug interaction
FFS: Fee-for-service
NDC: National drug code
NYS: New York State
MMC: Medicaid Managed Care
