# Supplementary material for: Prevalence of contraindicated combinations amid behavioral and mental health medications filled in a pediatric population
Source: BMC Prim Care. 2024 Jul 30;25:276. doi: 10.1186/s12875-024-02528-9 (PMC11289933; doi:10.1186/s12875-024-02528-9)
Supplement: Supplementary file 1 — Supplementary Material 1 [file 12875_2024_2528_MOESM1_ESM.docx]

**Supplementary Material (Submission ID 4a0f4479-32bf-43eb-b7dd-0212208dc381)**

**Prevalence of contraindicated combinations amid behavioral and mental health medications filled in a pediatric population**

**Appendix A. Detailed Methods.**

We defined BMH diagnoses using ICD-9 codes and two available schema: the Agency for Health Care Research and Quality’s Clinical Classification Software (AHRQ CCS) and several Healthcare Effectiveness Data and Information Set (HEDIS) behavioral health measures which required identifying people with BMH diagnoses through the HEDIS® value sets.^13,14^ The list of diagnoses was then reviewed and grouped into diagnostic classes by topic experts. Diagnostic classes included ADHD, anxiety, chronic and acute stress reactions, conduct disorder, depression, dementia, eating disorders, gender identity codes, learning, mood disorder, oppositional, peripartum mental disorders, personality disorder, pervasive, psychosis, sexual disorder, sleep disorder, somatoform, substance/alcohol use, suicide/self-injury, tic disorders, and other. BMH diagnoses were considered present if the ICD-9-CM codes for the above-noted diagnoses classes appeared in any position on any Medicaid claim or encounter in 2014.

Medications were classified as BMH medications using NDC codes and HEDIS®. Drug classes included: alpha-2 receptor agonists, central nervous system (CNS) stimulants, first generation antipsychotic medications, miscellaneous ADHD medications, miscellaneous antidepressants, monoamine oxidase inhibitors, phenothiazine antipsychotics, phenylpiperazine antidepressants, psychotherapeutic combinations, serotonin and norepinephrine reuptake inhibitor (SNRI) antidepressants, selective serotonin reuptake inhibitor (SSRI) antidepressants, second generation antipsychotic medications, tetracyclic antidepressants, tricyclic antidepressants, anti-anxiety, bipolar disorder, anticonvulsants, and benzodiazepine/anticholinergics/antispasmodics.

**Data Sources**

Our data come from 2014 NYS Medicaid pharmacy and FFS claim and MMC encounter data. We include patients enrolled at any point in the year and for any amount of time (no continuous enrollment inclusion criteria were included in the study). In this study population, 70% of Medicaid enrollees in 2014 were continuously enrolled the entire year. For those with a BMH medication, 85% of them were continuously enrolled the entire year. The authors opted not to apply a continuous enrollment criterion because to see all drug overlaps apparent in the data. The lack of continuous enrollment means that counts of DDI are potentially conservative. If the continuous enrollment criterion was applied, kids/overlaps that we identified through days supply could have been excluded (i.e., if a person was enrolled in January with a 90-day supply, and then they dropped enrollment in February, but picked it up again in March, our method would have identified an overlap with a drug dispensed in March).Those eligible for both Medicare and Medicaid (“dual eligible”) were excluded due to incomplete claim and encounter data.

Demographic information, including gender, race/ethnicity, county of residence, and age (calculated from date of birth), receipt of cash assistance (public assistance), insurance type (MMC vs FFS), and Medicaid eligibility category (those receiving Supplemental Security Income (SSI) or not), was obtained by linking member enrollment data. The patient’s county of residence was designated as urban if it mapped to USDA’s 2013 urban influence codes 1 or 2 (i.e., metropolitan areas with more than or fewer than 1 million residents); otherwise, the area was considered non-urban. Children receiving foster care services were identified using enrollment data provided by the NYS Office of Children and Family Services. SAS 9.4 was used to create Chi square contingency tables to determine demographic distribution of enrollees with BMH medication compared to those with CDPs.

Using Medicaid claim and encounter data, a relational database was created, linking tables comprised of member enrollment and demographic information, member BMD diagnosis data, and member BMH filled prescription data. One-day, 15-day, and 30-day time frames were constructed around each BMH prescription filled for each member and overlapping time frames were identified. An analysis was conducted for each BMH medication to determine 1) by how many members the medication was filled, and 2) for how many members was it filled concurrently with a contraindicated drug. SAS 9.4 was used to count members, their contraindicated drug pairs, and days of concurrent use. These data are presented in Tables 2 and 3. An analysis of the one-, 15-, and 30-day threshold for concurrency time frames recorded in the database, along with known side effects from the interaction found in Micromedex, are presented in Table 4.

Medicaid pharmacy claims data included NDC code, generic name/active ingredient, days’ supply, and date medication was filled at the pharmacy. Medications were grouped by active ingredient. Medication start date was the first date the drug was filled for the patient, while end date was calculated as start date plus days’ supply minus one. Concurrent use of contraindicated drugs was defined as overlapping fills of two or more contraindicated BMH medications for at least thirty days, allowing for a possible 32-day gap between consecutive start and end dates of the same medication. This threshold for concurrency time frame was selected after review of the literature and is consistent with current thinking about concurrency and included a clinical rationale to allow for medications to be titrated up or down when used concurrently in therapy transitions. Combination medications with two or more drug entities were excluded because they could not be categorized as multiple medications.

IBM Micromedex was used to identify which drug pairs were contraindicated.^13^ The Micromedex severity index indicates whether potential drug combinations or interactions are considered minor, moderate, major, or contraindicated. This paper focuses on contraindicated drug pairs or medications that are not intended to be used concurrently due to harm or a life-threatening situation. This most serious category includes combinations such as amitriptyline and ziprasidone, for which there is an increased risk of QT-interval prolongation and serotonin syndrome (hypertension, hyperthermia, myoclonus, mental status changes). Concurrent use of paroxetine and pimozide for which there is an increased risk of pimozide toxicity including cardiotoxicity (QT prolongation, torsades de pointes, cardiac arrest) would also be considered a contraindicated drug pair. According to IBM Micromedex, all contraindicated drug pairs included in this analysis had “fair” to “excellent” documentation. These contraindicated drug pairs are contrasted with minor interactions where clinical effects are limited, such as an increase in the frequency or severity of the side effects. For example, concurrent use of diazepam and fluoxetine for which the risk is higher serum concentrations of diazepam. We also omit from this report moderate and major interactions. Moderate interactions are more likely to require an alteration in therapy, such as the potential for concurrent use of alprazolam and sertraline to lead to psychomotor impairment and sedation. Major interactions may be life-threatening and/or require medical intervention to minimize or prevent serious adverse effects. An example would be concurrent use of desipramine and escitalopram with the potential for more serious impacts, such as increased desipramine exposure and increased risk of QT-interval prolongation.

| **Appendix B. BMH Medications Prescribed During Study Time Frame.** | |
| --- | --- |
| **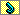BMH Medication (active ingredient*)** | **# of persons (<21 years) with BMH Rx filled** |
| Methylphenidate | 46,015 |
| Dextroamphetamine/amphetamine | 27,370 |
| Clonidine | 20,832 |
| Risperidone | 18,741 |
| Guanfacine | 18,307 |
| Sertraline | 12,680 |
| Fluoxetine | 10,626 |
| Lisdexamfetamine | 10,587 |
| Quetiapine | 8,778 |
| Aripiprazole | 8,616 |
| Dexmethylphenidate | 6,721 |
| Divalproex | 6,593 |
| Diazepam | 5,500 |
| Citalopram | 5,451 |
| Atomoxetine | 5,340 |
| Topiramate | 5,104 |
| Lamotrigine | 4,943 |
| Escitalopram | 4,697 |
| Trazodone | 4,594 |
| Bupropion | 4,115 |
| Clonazepam | 3,554 |
| Oxcarbazepine | 3,456 |
| Lithium | 3,351 |
| Olanzapine | 2,667 |
| Amitriptyline | 2,573 |
| Lorazepam | 2,355 |
| Gabapentin | 2,227 |
| Mirtazapine | 1,757 |
| Buspirone | 1,667 |
| Alprazolam | 1,546 |
| Paroxetine | 1,517 |
| Carbamazepine | 1,418 |
| Venlafaxine | 1,412 |
| Dextroamphetamine | 1,148 |
| Ziprasidone | 1,121 |
| Haloperidol | 848 |
| Chlorpromazine | 803 |
| Imipramine | 779 |
| Nortriptyline | 756 |
| Prochlorperazine | 727 |
| Fluvoxamine | 521 |
| Clozapine | 513 |
| Lurasidone | 405 |
| Duloxetine | 372 |
| Doxepin | 309 |
| Perphenazine | 261 |
| Paliperidone | 185 |
| Clomipramine | 157 |
| Fluphenazine | 135 |
| Asenapine | 81 |
| Desipramine | 47 |
| Chlordiazepoxide | 39 |
| Desvenlafaxine | 37 |
| Pimozide | 35 |
| Fluphenazine | 34 |
| Vilazodone | 34 |
| Clorazepate | 33 |
| Iloperidone | 25 |
| Loxapine | 21 |
| Vortioxetine | 19 |
| Thioridazine | 18 |
| Paliperidone | 16 |
| Trifluoperazine | 11 |
| Nefazodone | 10 |
| Thiothixene | 10 |
| Levomilnacipran | <6 |
| Protriptyline | <6 |
| Selegiline | <6 |
| Methamphetamine | <6 |
| Oxazepam | <6 |
| Amoxapine | <6 |

*The active ingredient listed includes all salt forms of the medication.
